# Tubular Overexpression of Angiopoietin-1 Attenuates Renal Fibrosis

**DOI:** 10.1371/journal.pone.0158908

**Published:** 2016-07-25

**Authors:** Sudhir Singh, Scott R. Manson, Heedoo Lee, Yeawon Kim, Tuoen Liu, Qiusha Guo, Julio J. Geminiani, Paul F. Austin, Ying Maggie Chen

**Affiliations:** 1 Division of Nephrology, Department of Internal Medicine, Washington University School of Medicine, St. Louis, MO, United States of America; 2 Division of Urology, Department of Surgery, Washington University School of Medicine, St. Louis, MO, United States of America; 3 Oncology Division, Department of Internal Medicine, Washington University School of Medicine, St. Louis, MO, United States of America; University Medical Center Utrecht, NETHERLANDS

## Abstract

Emerging evidence has highlighted the pivotal role of microvasculature injury in the development and progression of renal fibrosis. Angiopoietin-1 (Ang-1) is a secreted vascular growth factor that binds to the endothelial-specific Tie2 receptor. Ang-1/Tie2 signaling is critical for regulating blood vessel development and modulating vascular response after injury, but is dispensable in mature, quiescent vessels. Although dysregulation of vascular endothelial growth factor (VEGF) signaling has been well studied in renal pathologies, much less is known about the role of the Ang-1/Tie2 pathway in renal interstitial fibrosis. Previous studies have shown contradicting effects of overexpressing Ang-1 systemically on renal tubulointerstitial fibrosis when different engineered forms of Ang-1 are used. Here, we investigated the impact of site-directed expression of native Ang-1 on the renal fibrogenic process and peritubular capillary network by exploiting a conditional transgenic mouse system [Pax8-rtTA/(TetO)_7_ Ang-1] that allows increased tubular Ang-1 production in adult mice. Using a murine unilateral ureteral obstruction (UUO) fibrosis model, we demonstrate that targeted Ang-1 overexpression attenuates myofibroblast activation and interstitial collagen I accumulation, inhibits the upregulation of transforming growth factor β1 and subsequent phosphorylation of Smad 2/3, dampens renal inflammation, and stimulates the growth of peritubular capillaries in the obstructed kidney. Our results suggest that Ang-1 is a potential therapeutic agent for targeting microvasculature injury in renal fibrosis without compromising the physiologically normal vasculature in humans.

## Introduction

Renal fibrosis, characterized by interstitial myofibroblast activation and excessive matrix protein accumulation, is the final common pathway of chronic kidney disease (CKD) [1, 2]. Mounting evidence has demonstrated the pivotal role of the renal microvasculature in the development of interstitial fibrosis and CKD progression. Injury to the renal peritubular capillary network leads to capillary rarefaction, tubulointerstitial hypoxia, recruitment of inflammatory cells, and renal fibrosis [3–5]. Conversely, growth factors or cytokines with endothelial cell (EC) protective effects have been shown to ameliorate renal fibrosis in experimental models of renal fibrosis [6, 7]. Therefore, targeting the renal vasculature may be a promising therapeutic strategy to attenuate renal fibrosis.

Angiopoietins (Angs) are a family of vascular growth factors that bind to the EC specific tyrosine kinase receptor Tie2. Ang-1 and Ang-3 are agonists while Ang-2 and Ang-4 are antagonists for Tie2 [8–10]. Ang-1 is a 55-kDa glycoprotein secreted by proximal tubular cells and podocytes in the kidney [11], and it has a number of important functions in ECs as follows. 1. Ang-1 acts as a stabilization factor for new endothelial network formation through Ang-1-Tie2 paracrine signaling [12]. 2. Ang-1 is a strong anti-apoptotic survival factor in ECs [13]. 3. Ang-1 induces EC sprouting in *vitro* [14]. 4. Ang-1 stimulates EC migration in *vitro* [15]. In addition, Ang-1 has potent anti-inflammatory and anti-permeability effects [16, 17] and antagonizes the pro-angiogenic and pro-inflammatory actions of Ang-2 and vascular endothelial growth factor (VEGF) [18]. In summary, Ang-1 is a major physiological ligand for Tie2 and plays important roles in prenatal and postnatal vascular development. However, it is dispensable in mature and quiescent vessels [19].

Dysregulation of the Ang-1/Tie2 system has been shown to be an important feature of CKD in human patients [20]. Previous studies examining the therapeutic role of Ang-1 in renal tubulointerstitial disease have provided conflicting results. Kim et al. [21] showed that viral delivery of a chimeric form of Ang-1, cartilage oligomeric matrix protein-Ang-1 (COMP-Ang-1; a soluble and more potent Ang-1 variant than native Ang-1) preserves peritubular capillaries and decreases renal fibrosis and inflammation in a murine unilateral ureteral obstruction (UUO) fibrosis model. In contrast, Long et al. [22] found that administration of Ang-1*, a more soluble form of Ang-1, stabilizes peritubular capillaries in the chronic phase of folic acid (FA)-induced nephrotoxicity in mice, which is characterized by capillary loss and interstitial fibrosis. However, at the same time, the use of Ang-1* was accompanied by pro-fibrotic and pro-inflammatory effects. To determine the therapeutic potential of native Ang-1 in renal fibrosis, we have utilized a tetracycline-based binary conditional transgenic approach to overexpress Ang-1 specifically in mature renal tubules. We demonstrate here that enhanced tubular Ang-1 expression halts the progression of renal fibrosis in association with decreased renal inflammation and increased peritubular capillaries in a murine UUO model.

## Materials and Methods

### Generation of DOX-inducible TETAng-1 transgenic mice

All animal experiments conformed to the National Institutes of Health Guide for the Care and Use of Laboratory Animals and were approved by the Washington University Animal Studies Committee.

The (TetO)_7_/CMV-Ang-1-ZsGreen transgene contains the (TetO)_7_/CMV regulatory element (a gift from Jeffrey Miner, Washington University in St. Louis) driving a mouse Ang-1 cDNA, followed by an internal ribosome entry site (IRES), the ZsGreen cDNA and an SV40 polyadenylylation signal. Mouse Ang-1 was obtained from the plasmid pSPORT1-Ang-1 (ATCC, Manassas, VA) and cloned into the plasmid pIRES2-ZsGreen1 (Clontech, Mountain View, CA). The insert Ang-1-IRES-ZsGreen was subsequently cloned into the (TetO)_7_/CMV plasmid. TetAng-1 transgenic mice were identified by PCR using a primer pair (5’-TGCCACGTTGTGAGTTGGATAGTT-3’ and 5’-ACATGCAGTTCTCCTCCACGCT-3’) that amplifies a 593-bp fragment of the transgenic cDNA. The Pax8-rtTA (P8TA) transgenic mice were purchased from The Jackson Laboratory (JAX stock # 7176). P8TA mice were identified by PCR using a primer pair (5’-CCATGTCTAGACTGGACAAGA-3’ and 5’-CTCCAGGCCACATATGATTAG-3’) to generate an approximately 600-bp amplicon.

Transgenes were purified away from plasmid vector sequences and microinjected into the pronucleus of B6CBAF2/J single-celled embryos. To induce transgene expression, mice were fed TestDiet Modified LabDiet Rodent Diet 5001 containing 1500 ppm doxycycline (purchased from El-Mel, Inc, Florissant, MO) at three weeks of age for 8 weeks. Experimental mice remained on doxycycline food continuously, as did their single-transgene control littermates that were analyzed for comparison. At the end of experiments, mice were euthanized using intraperitoneal injection of ketamine/xylazine, followed by cervical dislocation. All procedures followed the American Veterinary Medical Association (AVMA) guide.

### Unilateral ureteral obstruction

A unilateral ureteral obstruction was created in 11-week-old bitransgenic P8TA/Ang-1 or single-transgene (Ang-1/+ or P8TA/+) control mice by surgically placing a microvascular clamp on the proximal ureter [23]. All procedures were approved by institutional review.

### Sirius Red staining

Paraffin sections of mouse kidneys were examined for renal fibrosis with the Picro-Sirius Red staining. Briefly, deparaffinized sections were treated with 0.1% Sirius Red in saturated picric acid (Sigma, St Louis, MO) and destained in 0.5% acetic acid. Collagen fibrils were stained and evaluated under light microscopy (Nikon) equipped with a polarizer. Ten randomly selected fields (magnification, x400) from cortex and medullar, respectively, in each kidney were evaluated and all images were captured by Olympus DP72 Capture Interface software. Ratio of Sirius red positive areas to whole areas in each field was calculated in percentages by Image J (NIH) software.

### Antibodies and reagents

Commercially available antibodies were obtained as follows: rabbit anti-mouse α-smooth muscle actin (α-SMA), rabbit anti-mouse collagen I, rat anti-mouse F4/80 (CI:A3-1), rabbit-anti-mouse CD31 antibodies were from Abcam (Cambridge, England), rabbit anti-mouse phospho-Smad 2/3(D27F4) and Smad2/3 (D7G7), rabbit anti-mouse GAPDH (D16H11) antibodies were from Cell Signaling (Danvers, Massachusetts), goat anti-Ang-1 was from Santa Cruz (Santa Cruz, CA), and horseradish peroxidase (HRP)-conjugated anti-mouse β actin antibody was from Sigma Aldrich. Alexa 594-conjugated secondary antibody was purchased from Molecular Probes (Carlsbad, CA). HRP-conjugated anti-rabbit and anti-goat secondary antibodies were from Santa Cruz. Histochoice was purchased from Amresco (Solon, OH).

### Immunofluorescence staining

Immunofluorescence staining on paraffin sections was performed as described previously [24]. For α-SMA, collagen I, CD31 and F4/80 staining, histochoice-fixed and paraffin-embedded sections were used. After dewaxing, the α-SMA, collagen I, CD31 antigens were retrieved by heating the slides in citrate buffer (pH 6.0) for 10 minutes at 100°C and the F4/80 antigen was stained without antigen retrieval. The kidney sections were blocked with 1% bovine serum albumin (BSA) for 30 minutes at room temperature, followed by overnight incubation with the individual primary antibody specified previously. The secondary antibody conjugated with Alexa 594 and Hoechst 33342 to stain nuclei were used to incubate for an hour. The slides were then mounted with anti-quench solution and visualized under a fluorescence microscope (Leica).

### Western blot analysis

The kidneys were extracted using RIPA buffer (Sigma) with protease and phosphatase inhibitor cocktails (Roche, Indianapolis, Indiana) and homogenized by sonication. The protein concentrations of kidney lysates were determined by Bio-Rad protein assay (Hercules, CA) using BSA as a standard. Denatured proteins were separated on sodium dodecyl sulfate polyacrylamide gel electrophoresis (SDS-PAGE) and then transferred to polyvinylidene difluoride membranes. Blots were blocked with 5% non-fat milk for 1 hour and then incubated overnight with primary antibodies. The membranes were washed with Tris-buffered saline/Tween buffer and incubated with the appropriate HRP–conjugated secondary antibodies. The proteins were then visualized in an x-ray developer using ECLplus detection reagents (GE, Pittsburgh, PA). To ensure equal protein loading, the same blot was stripped with stripping buffer (25mM glycine + 1% SDS, pH = 2.0) and then incubated with an anti-GAPDH or a HRP-conjugated mouse anti-mouse β-actin antibody. Relative intensities of protein bands were quantified using ImageJ analysis software.

### mRNA quantification by Real-Time PCR

Total RNA from sham-operated or UUO kidneys was extracted using the RNeasy kit (Qiagen, Valencia, CA) with subsequent DNase I treatment. 1 μg of kidney RNA was then reverse-transcribed using an RT-PCR kit (Superscript III; Invitrogen). Gene expression was evaluated by quantitative real-time PCR. One μl of cDNA was added to SYBR Green PCR Master Mix (Qiagen) and subjected to PCR amplification (one cycle at 95°C for 20 seconds, 40 cycles at 95°C for 1 second, and 60°C for 20 seconds) in an Applied Biosystems 7900HT Fast Real-Time PCR System (Life Technologies, Grand Island, NY) using mouse GAPDH as an internal control. Q-PCR was conducted in triplicate for each sample. The sequences of primers were following: mouse α-SMA forward: CCCACCCAGAGTGGAGAA, reverse: ACATAGCTGGAGCAGCGTCT; mouse collagen I forward: AGACATGTTCAGCTTTGTGGAC, reverse: GCAGCTGACTTCAGGGATG; mouse TNFα forward: ATGAGAAGTTCCCAAATGGCC, reverse: CCTCCACTTGGTGGTTTGCTA; mouse TGF-β1 forward: TGGAGCAACATGTGGAACTC, reverse: CAGCAGCCGGTTACCAAG; mouse MCP-1 forward: TTAAAAACCTGGATCGGAACCAA, reverse: GCATTAGCTTCAGATTTACGGGT; mouse ICAM-1 forward: AGCACCTCCCCACCTACTTT, reverse: AGCTTGCACGACCCTTCTAA; mouse Snail1 forward: CACACGCTGCCTTGTGTCT, reverse: GGTCAGCAAAAGCACGGTT; mouse Twist forward: CGGGTCATGGCTAACGTG, reverse: CAGCTTGCCATCTTGGAGTC; mouse GAPDH forward: TGTAGACCATGTAGTTGAGGTCA, reverse: AGGTCGGTGTGAACGGATTTG.

## Results

### Generation and characterization of inducible Ang-1 transgenic mice

**Fig 1:**
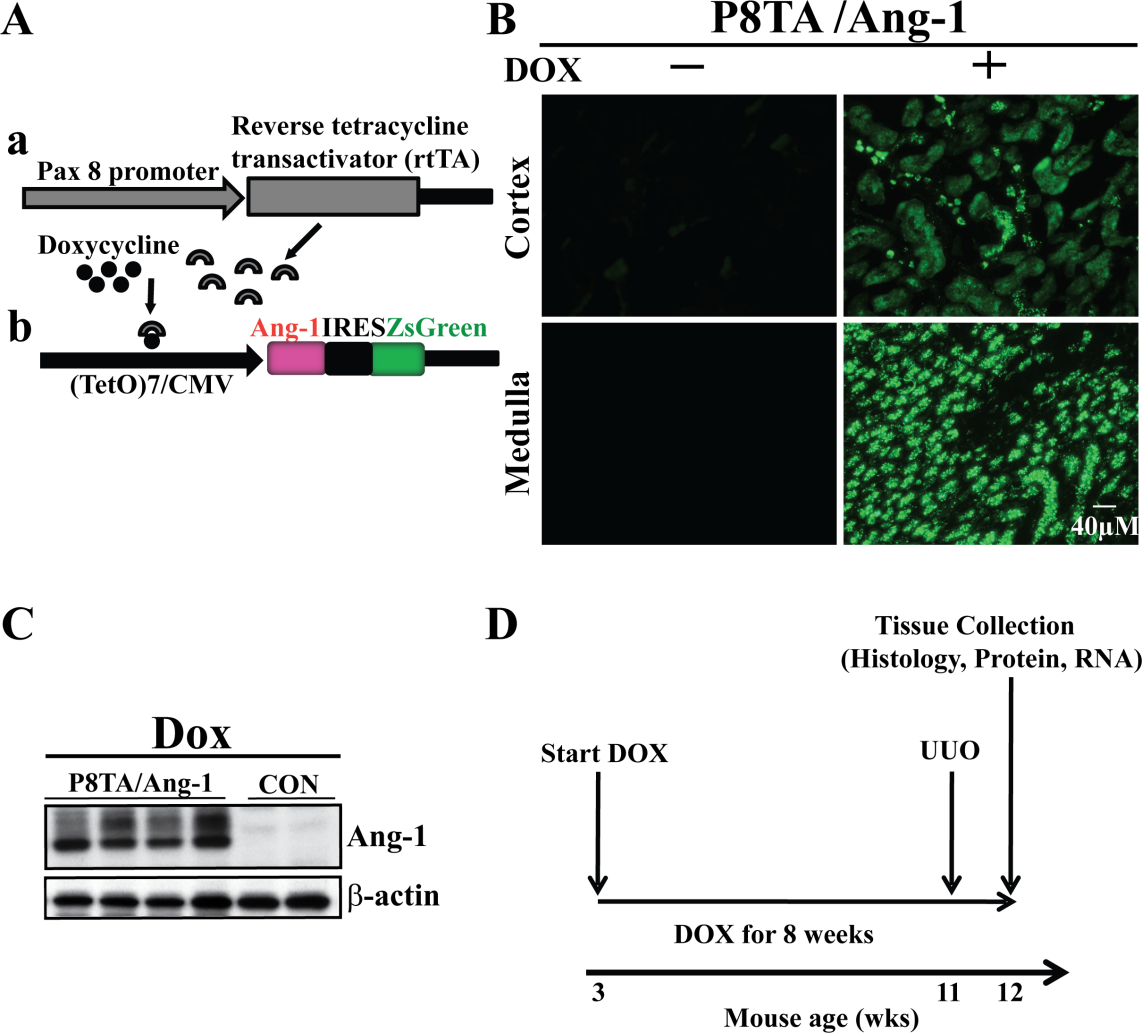
Transgene constructs and experimental plan. (A) Diagram of the two constructs used in the transgenic mouse lines for the generation of the reverse tetracycline-controlled transcriptional activator (rtTA) system (Tet-On system). **a.** The Pax8 promoter directs the expression of rtTA in renal tubular cells. **b.** The (TetO)_7_/CMV-Ang-1-ZsGreen transgene is induced by a doxycycline (DOX)-bound rtTA. (B) Frozen kidney sections from 11 week-old P8TA; TETANG-1 mice receiving regular or DOX food, respectively, at three weeks of age for 8 weeks. Green fluorescent protein (ZsGreen) was expressed in all segments of the renal tubules in double positive transgenic mice administered DOX. Scale bar, 40 μm. (C) Representative immunoblot of Ang-1 expression in whole kidney lysates from mice of the indicated genotypes after 8 weeks of DOX administration. (D) The experimental plan used for the UUO study. CON, control.

To investigate the effects of inducible tubular-specific Ang-1 upregulation on renal fibrosis, we generated doxycycline (DOX) inducible Ang-1 (TETAng-1) transgenic mice and utilized a reverse tetracycline-controlled transcriptional activator (rtTA) system (Tet-On system) (Fig 1A). In TETAng-1 transgenic mice, the mouse Ang-1 cDNA, followed by an internal ribosome entry site (IRES) and the ZsGreen cDNA, is controlled by a (TetO)_7_/CMV promoter containing 7 tetracycline-response elements (TREs) (Fig 1A). The IRES permits concurrent translation of Ang-1 and ZsGreen, the brightest known green fluorescent protein, from a single bicistronic mRNA. Meanwhile, Pax8-rtTA (P8TA) mice direct expression of rtTA in the entire renal tubular system (Fig 1A). In the presence of the tetracycline derivative DOX, rtTA binds to TRE and initiates transcription of the Ang-1 and ZsGreen cDNAs from the CMV promoter. Thus, ZsGreen expression is indicative of concurrent Ang-1 transgene expression.

Ten TETAng-1 transgenic lines were generated, and each was bred to the P8TA line. As a result, three genotypes were generated and abbreviated to P8TA/Ang-1, Ang-1/+ and P8TA/+ with a similar complements of background alleles. The offspring was induced with DOX at postnatal day 21 (P21) for 8 weeks, at which point Ang-1 expression in renal tubules was analysed by ZsGreen expression and Western blot (WB) with an anti-Ang-1 antibody. ZsGreen expression was strongly induced only in cortical and medullary renal tubules of P8TA/Ang-1 kidneys in the presence of DOX, whereas its expression was not detected in P8TA/Ang-1 kidneys in the absence of DOX (Fig 1B) or in Ang-1/+ or P8TA/+ kidneys in the presence of DOX (data not shown). Furthermore, as demonstrated by WB, kidney lysates from P8TA/Ang-1 mice that were treated with DOX for 8 weeks showed significantly increased Ang-1 levels compared to those from mice expressing a single Ang-1 or P8TA transgene that were treated with DOX for 8 weeks (Fig 1C) or from P8TA/Ang-1 mice in the absence of DOX (data not shown). Consistent with a previous report [25], both glycosylated and unglycosylated forms of Ang-1 were observed in kidney tissues. For our studies, we selected the transgenic line that exhibited the highest inducible expression of the transgene without basal leaky expression. Histologic examination revealed no conspicuous histological changes in P8TA/Ang-1 kidneys compared with control single-transgene kidneys that had been exposed to DOX for 8 weeks (S1 Fig). Body weight was also not different among the above-mentioned groups (data not shown). We therefore reasoned that this inducible transgenic strategy could be used as a means of manipulating Ang-1 levels in models of renal interstitial fibrosis.

### Renal tubular overexpression of Ang-1 ameliorates renal fibrosis

**Fig 2:**
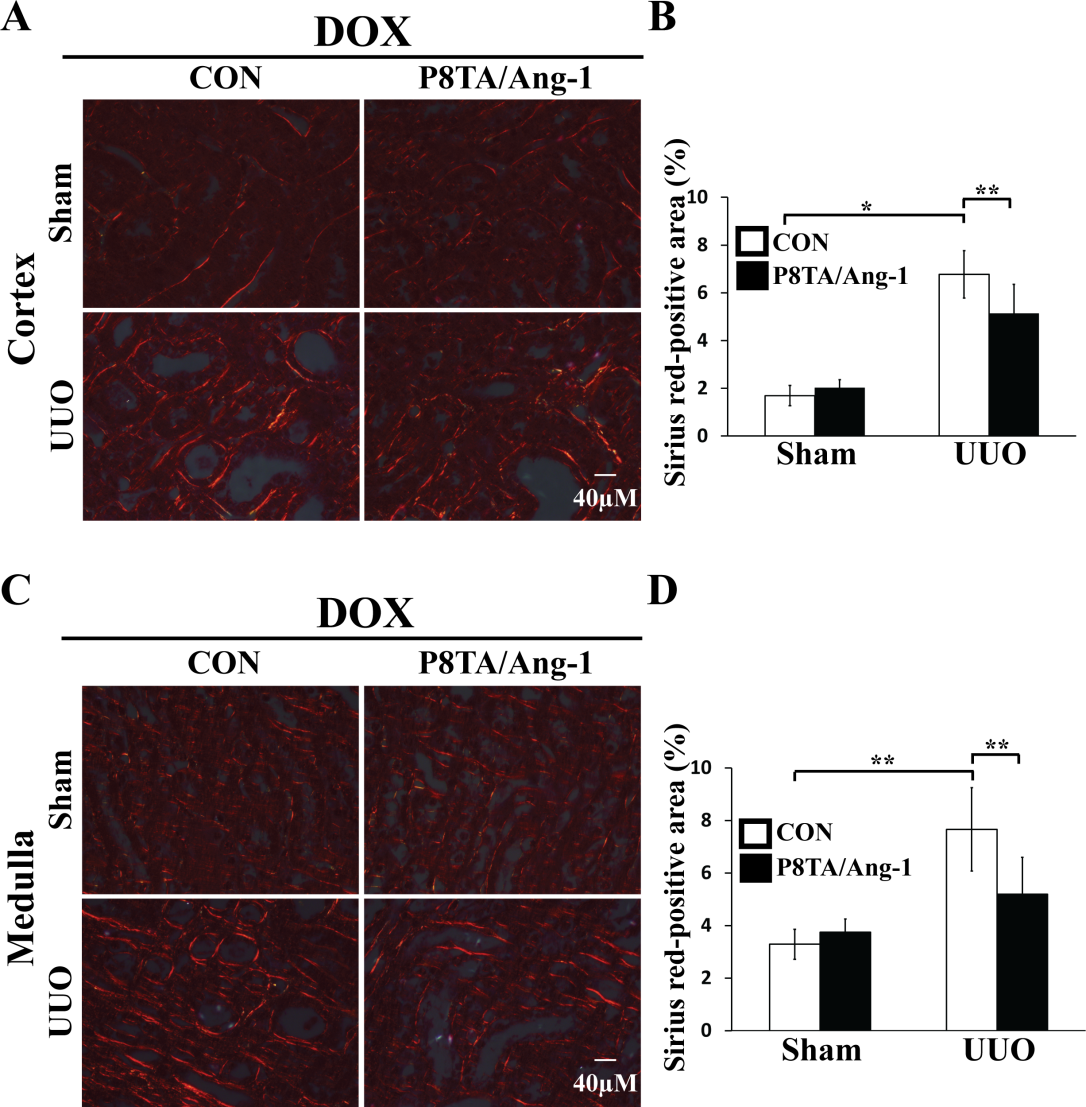
Targeted overexpression of Ang-1 in renal tubules attenuates renal interstitial fibrosis after UUO. Bitransgenic P8TA/Ang-1 or single-transgene control mice (Ang-1/+ or P8TA/+) underwent either sham operation or 7 days of obstruction (n = 6 mice/group) after 8 weeks of DOX administration. Fibrosis of kidney cortex (A) and medulla (C) was analyzed by Sirius Red staining under polarized light. Scale bars, 40 μm. Morphometric quantification of Sirius Red-positive area in cortex (B) or medulla (D), respectively, from sagittal kidney sections in the indicated groups (n = 6 mice/group). Ten photographs / kidney were uniformly taken in the cortex or medulla, respectively, at x400 magnification. Composite microscopy images were analyzed using Image J software. * P < 0.05; ** P< 0.001 by ANOVA. CON, control.

To evaluate the effects of renal tubular expression of Ang-1 on interstitial fibrosis, we utilized a murine model of obstruction-induced renal injury. In this model, a UUO is created in mice by placing a microvascular clamp on the proximal ureter [26]. UUO results in the development of fibrotic changes in glomeruli and the interstitium that are characterized by tubular dilation and interstitial collagen accumulation. From P21, P8TA/Ang-1, P8TA/+ or Ang-1/+ mice were fed DOX for 8 weeks, at which point these mice underwent either sham operation or 7 days of UUO. These mice were studied in detail when they were 12 weeks old (i.e., 9 weeks after DOX induction) (Fig 1D). Kidney sections were stained with Picro-Sirius Red, which stains fibrils containing type I and III collagens. As shown in Fig 2, control mice (Ang-1/+ or P8TA/+) that were treated with DOX and subjected to UUO exhibited marked interstitial fibrosis, as indicated by positive Sirius Red staining in both cortex (Fig 2A and 2B) and medulla (Fig 2C and 2D) in comparison to sham-operated, DOX-treated control mice. In contrast, renal tubular overexpression of Ang-1 in P8TA/Ang-1 DOX mice significantly reduced the Sirius Red-positive area in both the cortex (Fig 2A and 2B) and medulla (Fig 2C and 2D) of obstructed kidneys when compared with control mice subjected to UUO. These results convincingly demonstrate that targeted overexpression of Ang-1 in renal tubules mitigates renal interstitial fibrosis.

### Targeted tubular Ang-1 upregulation inhibits renal expression of TGF-β1, collagen I and α-SMA

**Fig 3:**
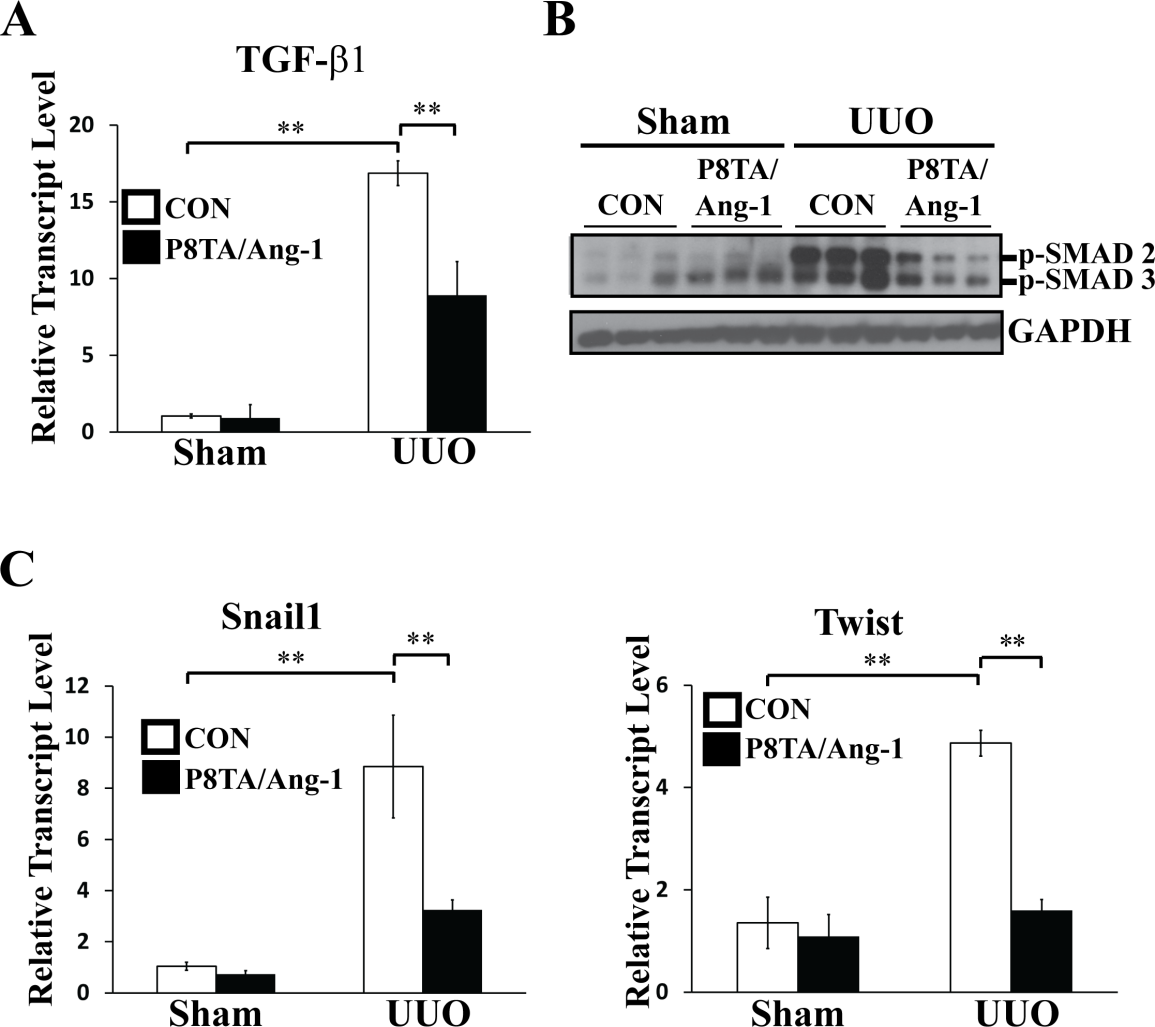
Targeted overexpression of Ang-1 in renal tubules downregulates TGF-β1 signaling. P8TA/Ang-1 and control (Ang-1/+ or P8TA/+) mice underwent either sham operation or 7 days of UUO (n = 6 mice/group) after DOX treatment for 8 weeks. Kidneys were harvested for both RNA and protein analyses at 12 weeks of age. (A) Renal TGF-β1 transcript level was assessed by quantitative RT-PCR in the indicated groups. TGF-β1 expression was normalized as a ratio to mouse GAPDH, and the average TGF-β1/GAPDH ratio in sham-operated control mice was set as 1. Mean ± SD; ** P< 0.001 by ANOVA. (B) Representative immunoblot analysis for phospho-Smad 2/3 and GAPDH proteins in kidney lysates from the indicated groups. (C) Quantitative RT-PCR showed relative transcript levels of Snail1 and Twist in kidneys from the indicated groups. Absolute levels were normalized first to those of GAPDH and then to the levels in the sham-operated control kidneys. Mean ± SD; ** P< 0.001 by ANOVA. CON, control.

To further explore the molecular mechanisms underlying the anti-fibrotic effects of targeted Ang-1 expression in renal tubules, we examined the renal expression of transforming growth factor β1 (TGF-β1) in P8TA/Ang-1 DOX and control (Ang-1/+ or P8TA/+) DOX mice that underwent either sham operation or 7 days of UUO. It is known that TGF-β1 plays a central role in the pathogenesis of renal injury. Upon TGF-β1 stimulation, Smad2 and Smad3 are recruited to type I TGF-β1 receptors and phosphorylated. Phosphorylated Smads then form heteromeric complexes with the common partner Smad4 and translocate into the nucleus, where they control the transcription of TGF-β-responsive genes [27–29]. Real-time quantitative PCR (q-PCR) demonstrated that renal TGF-β1 mRNA levels were significantly upregulated in obstructed control DOX kidneys following 7 days of UUO, as compared to levels in sham-operated control DOX kidneys (Fig 3A). The upregulation of renal TGF-β1 mRNA levels in UUO kidneys was attenuated by transgenic tubular Ang-1 expression in P8TA/Ang-1 DOX kidneys (Fig 3A). Furthermore, the subsequent increase in phosphorylation of Smad 2/3 in UUO control DOX kidneys, as compared to sham-operated kidneys, was attenuated by induced tubular Ang-1 upregulation in UUO P8TA/Ang-1 DOX kidneys (Fig 3B).

It has been proposed that microvascular pericytes, perivascular fibroblasts, tubular epithelial cells, and more recently ECs can undergo mesenchymal transition to form scar-producing myofibroblasts [30–33]. TGF-β signaling has been shown to induce epithelial-mesenchymal transition (EMT) of tubular epithelial cells, thus contributing to tubulointerstitial fibrosis [34]. TGF-β induces EMT via Smad-dependent and -independent signaling pathways, and transcription factors identified downstream of TGF-β signaling include Snail/Slug, Twist, ZEB1 and ZEB2 [35, 36]. As shown in Fig 3C, transcript levels of Snail1 and Twist were significantly increased in UUO control DOX kidneys following 7 days of obstruction compared with sham-operated kidneys. Moreover, tubular overexpression of Ang-1 in UUO P8TA/Ang-1 DOX kidneys mitigated the obstruction-induced increase of Snail1 and Twist mRNA levels.

**Fig 4:**
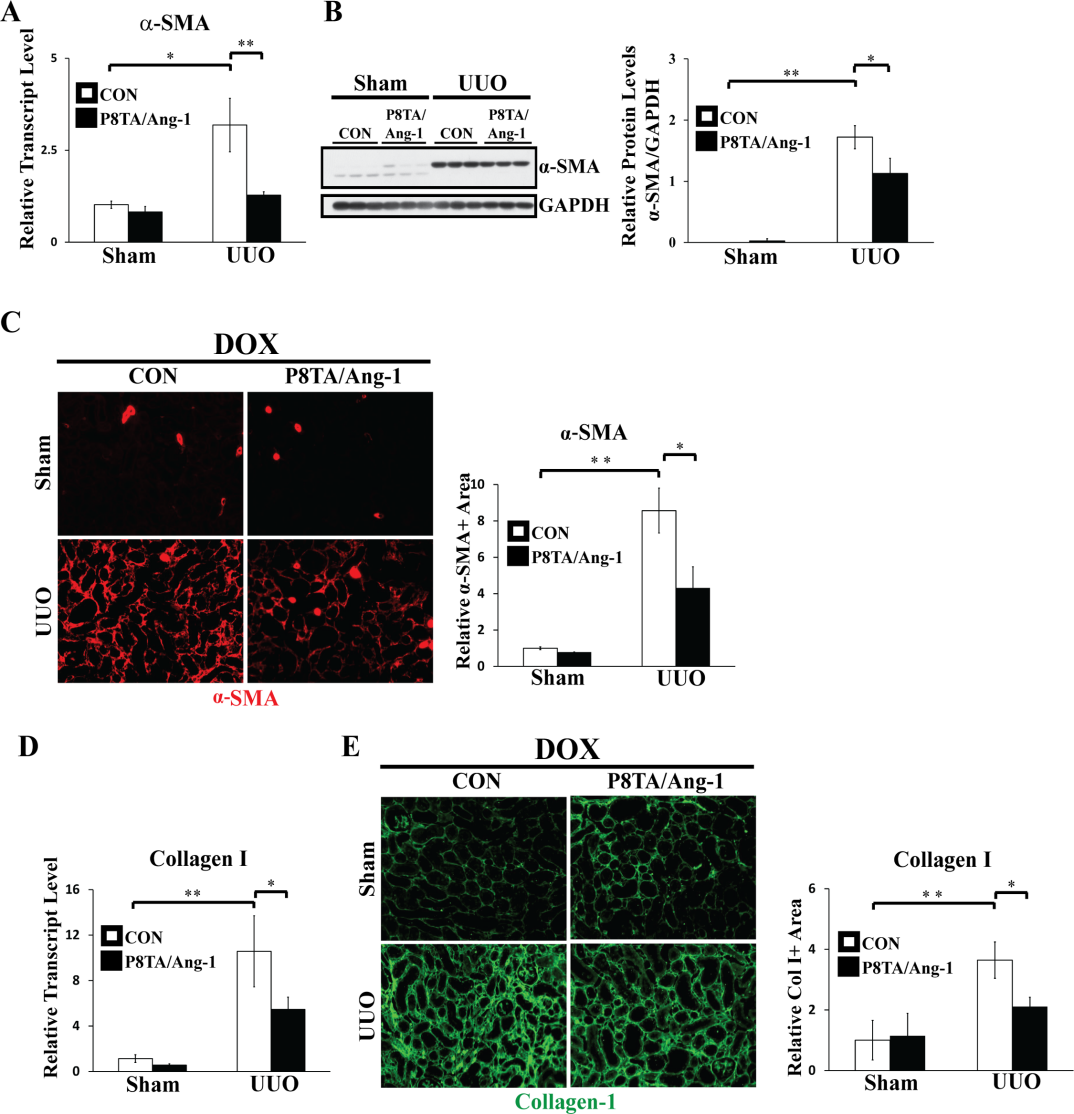
Targeted overexpression of Ang-1 in renal tubules ameliorates renal pro-fibrotic responses. DOX-treated P8TA/Ang-1 and control (Ang-1/+ or P8TA/+) mice underwent either sham operation or 7 days of UUO (n = 6 mice/group). Kidneys were harvested for both RNA, protein and IF analyses at 12 weeks of age. (A and D) Quantitative RT-PCR showed relative transcript levels of α-SMA (A) and collagen I (D) in sham-operated or UUO kidneys from the indicated genotypes. Absolute levels were normalized first to those of GAPDH and then to the levels in the sham-operated single transgene control kidneys. * P < 0.05; ** P< 0.001 by ANOVA. (B) Whole kidney lysates from the indicated groups were analyzed by WB for levels of α-SMA and GAPDH. Quantification of α-SMA was normalized to GAPDH in kidney lysates. The average α-SMA/GAPDH ratio in sham-operated control mice was set as 1. Mean ± SD; * P < 0.05 and ** P< 0.001 by ANOVA. (C and E) IF staining with quantification for α-SMA (red) (C) or collagen I (green) (E) on paraffin kidney sections from the indicated groups. Original magnifications: x200. Morphometric quantification of α-SMA-(C) or collagen I-(E) positive area in cortex from sagittal kidney sections in the indicated groups (n = 6 mice/group). Five photographs / kidney were uniformly taken in the cortex at x200 magnification. Composite microscopy images were analyzed using Image J software. The α-SMA- or collagen I-positive area in sham-operated control mice was set as 1. Mean ± SD; * P < 0.05; ** P< 0.001 by ANOVA. CON, control.

Myofibroblasts are characterized by expression of α-smooth muscle actin (α-SMA) and production of extracellular matrix (ECM) components including type I and III collagens [32, 37]. TGF-β1 can stimulate both myofibroblast activation and collagen production [38]. We thus hypothesized that renal tubular-specific overexpression of Ang-1 can also attenuate the upregulation of α-SMA and collagen I in obstructed kidneys. Indeed, q-PCR showed a significant decrease in renal transcript levels of α-SMA and collagen I in UUO P8TA/Ang-1 DOX kidneys when compared to UUO control DOX kidneys after 7 days of obstruction (Fig 4A and 4D). Consistent with the q-PCR data, WB demonstrated a significant reduction of renal α-SMA protein levels in obstructed kidneys from enhanced tubular expression of Ang-1 in P8TA/Ang-1 DOX mice compared to control DOX mice (Fig 4B). Furthermore, immunofluorescence (IF) staining of α-SMA (Fig 4C) and collagen I (Fig 4E) confirmed that tubular-specific overexpression of Ang-1 in UUO P8TA/Ang-1 DOX kidneys significantly inhibited upregulation of α-SMA and collagen I in obstructed control kidneys. Collectively, these data clearly indicate that renal tubular overexpression of Ang-1 attenuates TGF-β signaling and fibrogenesis.

### Targeted tubular Ang-1 overexpression reduces UUO-induced renal inflammation

**Fig 5:**
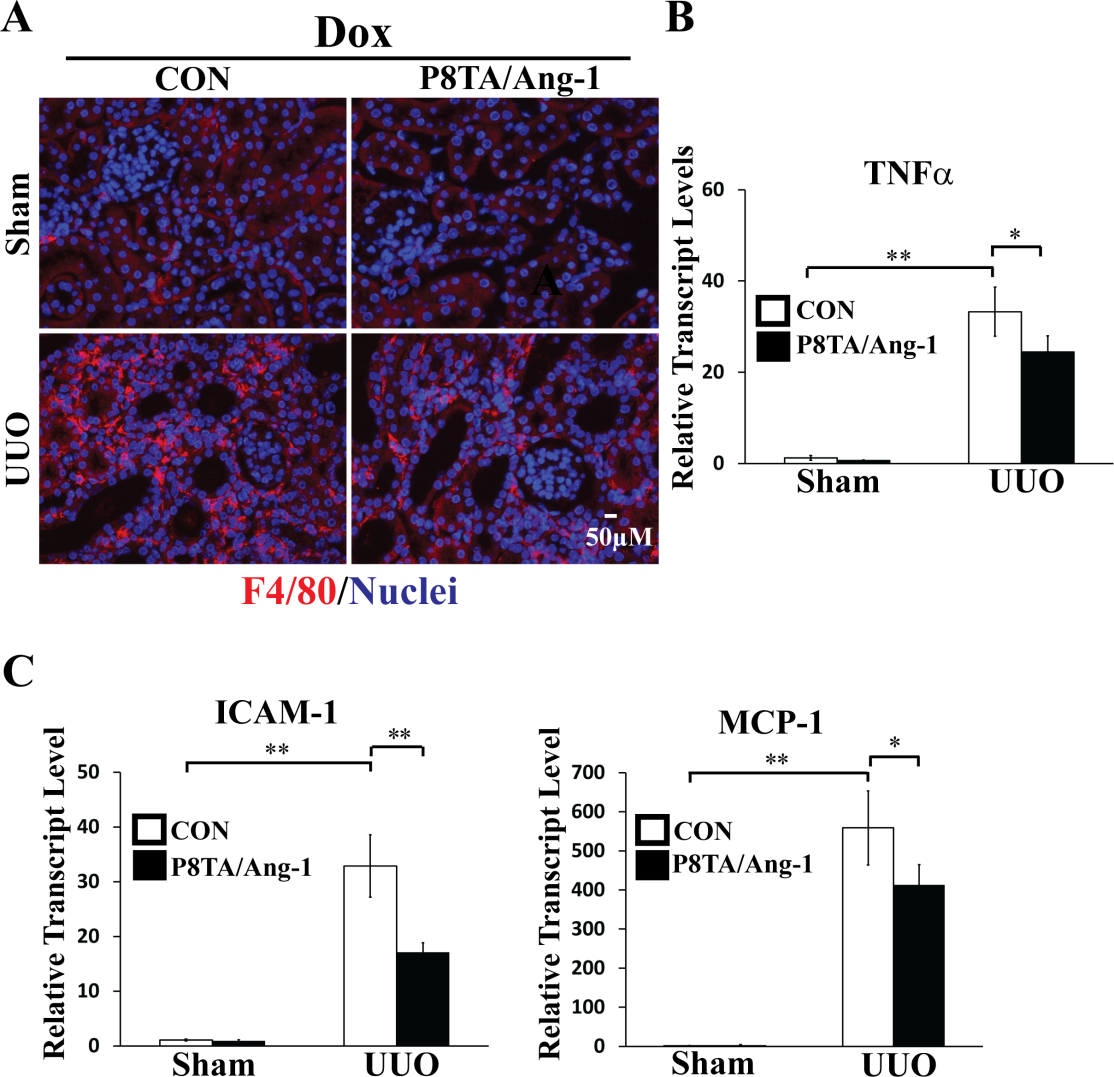
Targeted tubular Ang-1 upregulation suppresses UUO-induced inflammation. P8TA/Ang-1 and control (Ang-1/+ or P8TA/+) mice exposed to 8 weeks of DOX underwent either sham operation or 7 days of UUO (n = 6 mice/group). Kidneys were harvested for both IF staining and mRNA quantification at 12 weeks of age. (A) IF staining for F4/80 (red) on paraffin kidney sections from the indicated groups. Nuclei were counterstained with Hoechst 33342 (blue). Scale bar, 50 μm. (B) Renal TNFα transcript level was assessed by q-RT-PCR in the indicated groups. TNFα expression was normalized as a ratio to mouse GAPDH, and the average TNFα/GAPDH ratio in sham-operated control mice was set as 1. Mean ± SD; * P < 0.05 and ** P< 0.001 by ANOVA. (C) Quantitative RT-PCR showed relative transcript levels of ICAM-1 and MCP-1 in kidneys from the indicated groups. Absolute levels were normalized first to those of GAPDH and then to the levels in the sham-operated control kidneys. Mean ± SD; * P < 0.05 and ** P< 0.001 by ANOVA. CON, control.

The homing of inflammatory macrophages to renal tissues has been identified as a potent mechanism in fibrosis progression [39, 40], and macrophage-derived factor TNFα has been shown to be a key pathogenic regulator of UUO-induced renal fibrosis [41–43]. We next determined whether renal overexpression of Ang-1 can inhibit inflammation in obstructed kidneys. Indeed, renal macrophage infiltration, as identified by IF staining for the F4/80 antigen, was easily detectable in obstructed control kidneys after 7 days of injury (Fig 5A and S2 Fig). In contrast, this increase in F4/80-positive macrophage infiltration was attenuated by tubular Ang-1 overexpression in UUO P8TA/Ang-1 DOX kidneys (Fig 5A and S2 Fig). The subsequent increase in TNFα transcript levels in UUO control DOX kidneys was also reduced by renal Ang-1 overexpression in UUO P8TA/Ang-1 DOX kidneys (Fig 5B). Consequently, transgenic tubular Ang-1 expression ameliorated the transcriptional induction of intercellular adhesion molecule-1 (ICAM-1) and monocyte chemoattractant protein-1 (MCP-1), which are target inflammatory gene and chemokine of TNFα and its downstream transcriptional factor NFκB, in obstructed kidneys (Fig 5C). Taken together, these results demonstrate that transgenic Ang-1 expression in renal tubules mitigates obstruction-induced inflammation.

### Effect of tubular Ang-1 overexpression on renal microvasculature

**Fig 6:**
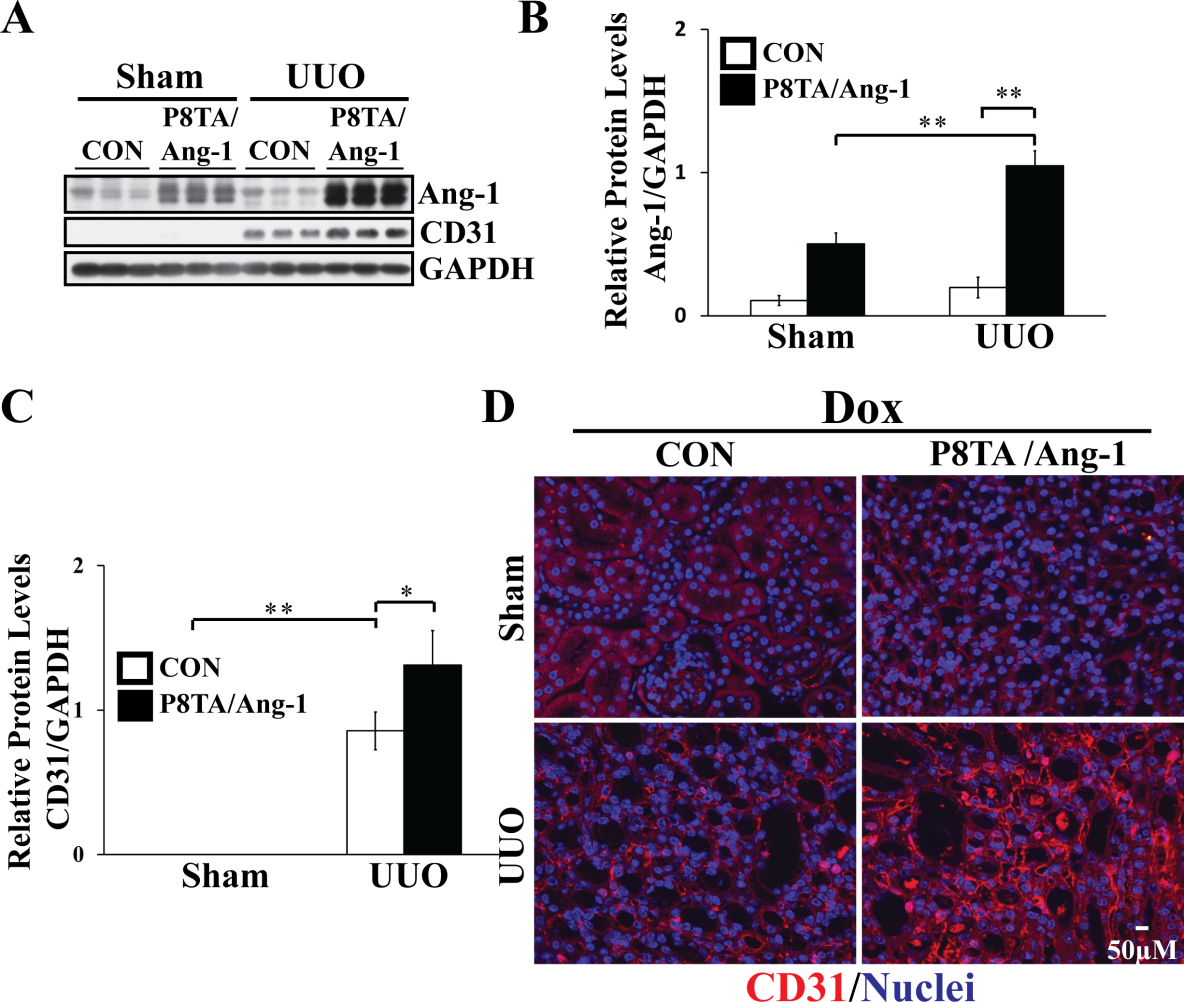
Tubular-directed Ang-1 overexpression promotes the growth of peritubular capillary network after UUO. (A) P8TA/Ang-1 and control (Ang-1/+ or P8TA/+) mice underwent either sham operation or 7 days of UUO (n = 6 mice/group) after DOX treatment for 8 weeks. Kidneys were harvested and lysates were analyzed by WB for levels of Ang-1, CD31, and GAPDH. Quantification of (B) Ang-1 or (C) CD31 normalized to GAPDH in kidney lysates of the indicated groups. The average Ang-1/GAPDH or CD31/GAPDH ratio in sham-operated control mice was set as 1. Mean ± SD; * P < 0.05 and ** P< 0.001 by ANOVA. (D) IF staining for CD31 (red) on paraffin kidney sections from DOX-treated P8TA/Ang-1 and control (Ang-1/+ or P8TA/+) mice undergoing either sham operation or 7 days of UUO (n = 6 mice/group). Nuclei were counterstained with Hoechst 33342 (blue). Scale bar, 50 μm. CON, control.

The UUO kidney injury model is characterized by an early angiogenic response and increased capillary density persisting through day 7 and microvascular rarefaction beginning at about day 10 after injury [44]. We further assessed the impact of site-directed Ang-1 overexpression on the peritubular capillary compartment after obstruction-induced injury. Consistent with previous studies, there was a significant increase in peritubular capillary density as assessed by WB (Fig 6A and 6C) and IF staining (Fig 6D and S3 Fig) for CD31, an EC marker, on day 7 following UUO-induced injury in control kidneys compared to sham-operated kidneys. Moreover, on day 7 of UUO, there was a more robust peritubular CD31 expression in response to enhanced tubular expression of Ang-1 in UUO P8TA/Ang-1 DOX kidneys compared to UUO control DOX kidneys (Fig 6A, 6C and 6D). In sham-operated mice, CD31 expression was also increased in P8TA/Ang-1 DOX kidneys compared with control DOX kidneys with much longer exposure time (data not shown). In addition, it appears that the obstruction-induced injury enhanced renal Ang-1 expression in UUO kidneys versus sham-operated kidneys in P8TA/Ang-1 DOX mice (Fig 6A and 6B). Together, these data suggest that tubular Ang-1 overexpression induces angiogenesis and increases the renal microvasculature in conjunction with the suppression of renal fibrosis.

## Discussion

Our study demonstrates for the first time that conditional overexpression of native Ang-1 in renal tubules attenuates renal interstitial fibrosis, dampens renal inflammation, and promotes the growth of the renal peritubular microvasculature after obstruction-induced injury. Our findings are in accord with a previous report that Ang-1 deficiency exaggerates the fibrotic response to injury or microvascular stress [19].

Ang-1 consists of a carboxy-terminal fibrinogen-like domain that mediates its differential effects on Tie2 phosphorylation, a central coiled-coil domain that oligomerizes these fibrinogen-like domains, and a short amino (N)-terminal domain that clusters these oligomers into multimers [45]. When Ang-1 was administered systemically by adenoviral transduction of hepatocytes, the central coiled-coil and N-terminal superclustering domains, which are responsible for the modular and multimeric structure of Ang1, led to protein aggregation and insolubility in circulation. To overcome these problems, several engineered Ang-1 variants were generated in previous studies. In COMP-Ang-1, the N-terminal portion of Ang-1 was replaced with the short coiled-coil domains of COMP. In Ang-1*, the N-terminus of Ang-1 incorporated a portion of Ang-2 together with a mutated Cys-245 in the central coiled-coil domain [46]. These recombinant variants are different from native Ang-1 in their biological functions. For example, COMP-Ang-1 is more potent than native Ang-1 in phosphorylating Tie2 [46] and also induces larger vessels and increases blood flow [47], mimicking the activating mutation of Tie2 found in inherited venous malformations [48]. Ang-1* with its N-terminus more closely resembling Ang-2 could induce an inflammatory response. These different engineered forms of Ang-1 likely account for the contradicting effects of Ang-1 observed in murine renal fibrosis models [21, 22]. In this study, we avoided these limitations by successfully employing a targeted transgenic strategy to demonstrate the protective effects of native Ang-1 in renal fibrosis *in vivo*.

TGF-β1/Smad signaling is the central pathway for myofibroblast activation and differentiation [49, 50]. In progressive fibrogenesis, TGF-β/Smad and TNFα pathways amplify each other to drive fibrosis and inflammation [51–53]. Here, we showed that tubular overexpression of Ang-1 counteracts both of these UUO-induced signaling pathways. These results confirm prior studies suggesting that the actions of Ang-1 are anti-fibrotic and anti-inflammatory. Our data have also demonstrated that Ang-1 suppresses renal TGF-β-mediated EMT. In addition, ECs represent a unique target for TGF-β. Recently it has been shown that partial deletion of TGF-β receptor II in the endothelium reduces endothelial-to-mesenchymal transition (EndoMT) and blunts renal interstitial fibrosis [54]. To further dissect the cellular mechanisms underlying the anti-fibrotic effects of Ang-1, we will employ coculture of ZsGreen-labelled primary tubular cells overexpressing Ang-1 with primary kidney microvascular ECs in 3D collagen gel to study the interaction between Ang-1/Tie2 signaling and TGF-β pathway in endothelial cells. In addition, fibrosis and inflammation form a self-perpetuating malicious cycle to accelerate progression of fibrosis. An important component of the inflammatory response is the migration of leukocytes from the blood vessel into the kidney tissue. In vitro, Ang-1 decreases permeability between endothelia by strengthening endothelial cell-cell junctions [16, 55, 56]. In vivo, transgenic mice overexpressing Ang-1 in the skin reveal that skin vessels are resistant to leakage caused by inflammatory agents [57]. It has also been shown that Ang-1 can restore poorly remodeled and leaky vessels [58]. Finally, activation of Tie2 by Ang-1 can inhibit TNFα-induced leukocyte capillary transmigration [16] by blocking NF-κB that controls expression of inflammatory genes such as ICAM1 or vascular cell adhesion molecule-1 (VCAM-1) in endothelial cells [59]. We also would like to point out that the actions of Ang-1 are not exclusively Tie-2 dependent. Ang-1 can also bind to and activate endothelial integrins such as α5β1 to mediate its bioactivity in the absence of Tie2 [60, 61]. Future experiments will be required to understand whether integrin complexes are stimulated by Ang-1 in renal fibrosis.

We also found that after 7 days of UUO, CD31-positive peritubular ECs in UUO kidneys were increased compared to sham-operated kidneys from single-transgene control mice, which is consistent with previous reports [21, 44]. Moreover, the increase in peritubular capillary density was accentuated by tubular overexpression of Ang-1, suggesting that Ang-1 can stimulate angiogenesis and growth of peritubular capillaries following UUO. This effect might be due to Ang-1 directly enhancing proliferation [47] or preventing endothelial apoptosis [62], which warrants further investigation. Moreover, we plan to use a recently reported fluorescence microangiography technique [63] to visualize and quantify the difference in peritubular capillary perfusion between control and Ang-1 overexpressing mice.

The molecular mechanisms controlling developmental and pathogenic angiogenesis are complex and involve a coordinated effort among endothelial growth factors and their receptors. VEGF and angiopoietin are two major endothelial signaling pathways modulating angiogenesis. Both upregulation and downregulation of VEGF in podocytes [64, 65] or renal tubules [66, 67] at any time point have profound effects on the glomerular filtration barrier or tubulointerstitial compartments, respectively. For instance, postnatal overexpression of VEGF in renal tubules of Pax8-rtTA/(tetO)_7_VEGF mice causes capillary-rich interstitial fibrosis, cyst formation and disruption of glomerular architecture [67]. On the other hand, embryonic or postnatal ablation of tubular VEGF results in the formation of a smaller kidney with a striking reduction in the peritubular capillary density and polycythemia due to increased renal erythropoietin production [66]. In contrast, although the Ang-1/Tie2 pathway is critical for vessel development, as conventional Ang-1 or Tie2 knockout mice exhibit lethality between E9.5 and E12.5 with similar abnormal vascular phenotypes [9, 68], global deletion of Ang-1 from E13.5 onward does not impair vascular integrity or increase permeability in mature, quiescent vascular beds [19]. Our results showed that expression of excess Ang-1 in renal tubules starting at three weeks of age does not result in overt tubular or capillary structural abnormality. These data strongly suggest that the Ang-1/Tie2 signaling pathway is an appealing therapeutic target to protect against renal tubulointerstitial fibrosis since manipulation may selectively control microvascular injury without compromising the normal vascular function.

## Supporting Information

S1 Fig No obvious histological abnormality was observed in P8TA/Ang-1 kidneys compared with control kidneys after 8 weeks of DOX administration. The Ang-1 overexpression in P8TA/Ang-1 kidneys was induced by DOX at 3 weeks of age. Scale bar, 40 μm.
(PDF)

S2 Fig Paraffin kidney sections from mice of the indicated groups were examined by immunofluorescence staining for F4/80 (red). Nuclei were counterstained with Hoechst 33342 (blue). Individual and merged images were shown as indicated. Scale bar, 250 μm. CON, control.
(PDF)

S3 Fig Paraffin kidney sections from the indicated groups were stained for CD31 (red). Nuclei were counterstained with Hoechst 33342 (blue). Individual and merged images were shown as indicated. Scale bar, 250 μm. CON, control.
(PDF)
