# Supplementary figures and images for: Tubular Overexpression of Angiopoietin-1 Attenuates Renal Fibrosis
Source: PLoS One. 2016 Jul 25;11(7):e0158908. doi: 10.1371/journal.pone.0158908 (PMC4959721; doi:10.1371/journal.pone.0158908)

# DOX

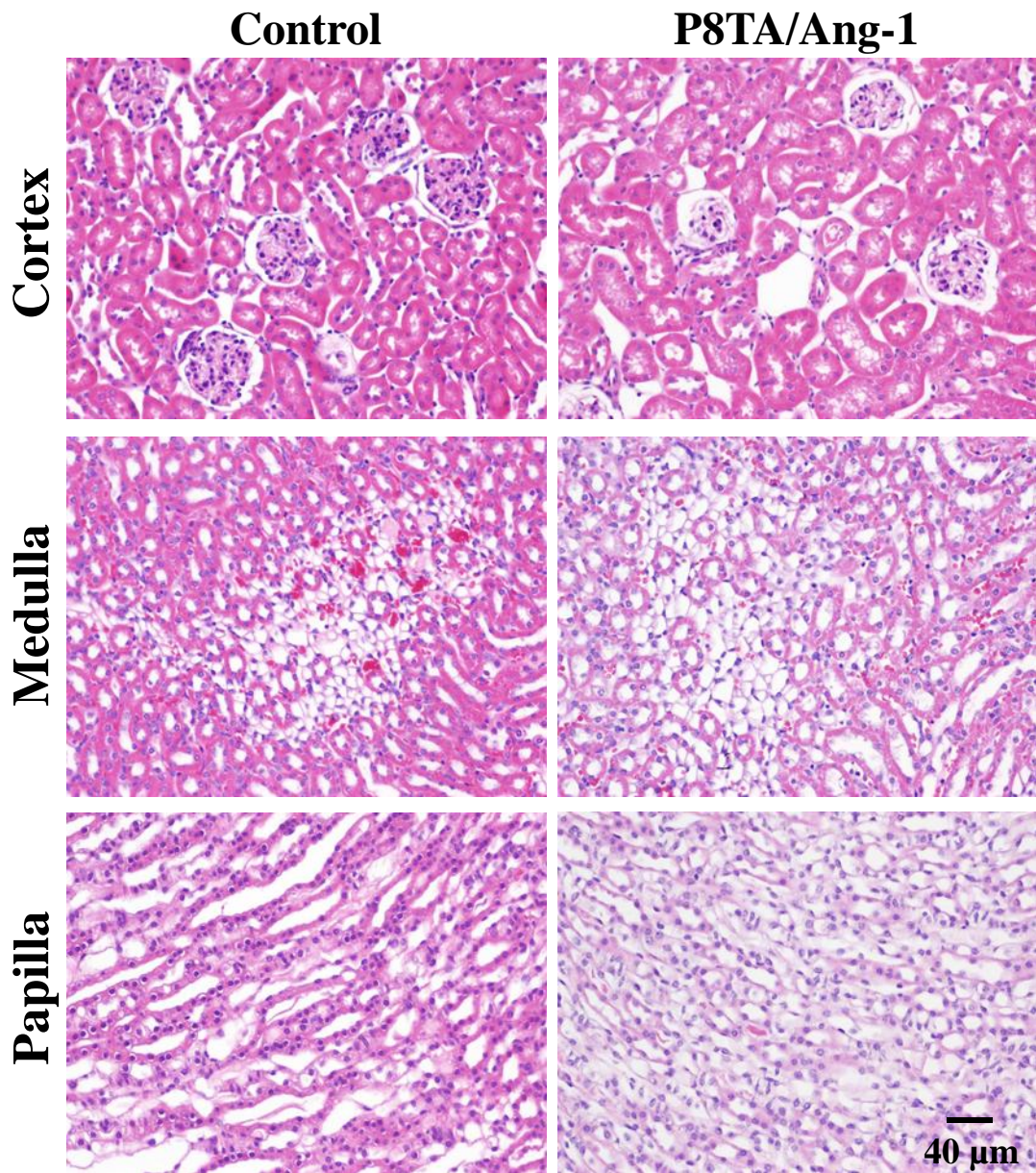

S1 Fig

Supplement: S1 Fig — The Ang-1 overexpression in P8TA/Ang-1 kidneys was induced by DOX at 3 weeks of age. Scale bar, 40 μm. (PDF) [file pone.0158908.s001.pdf]

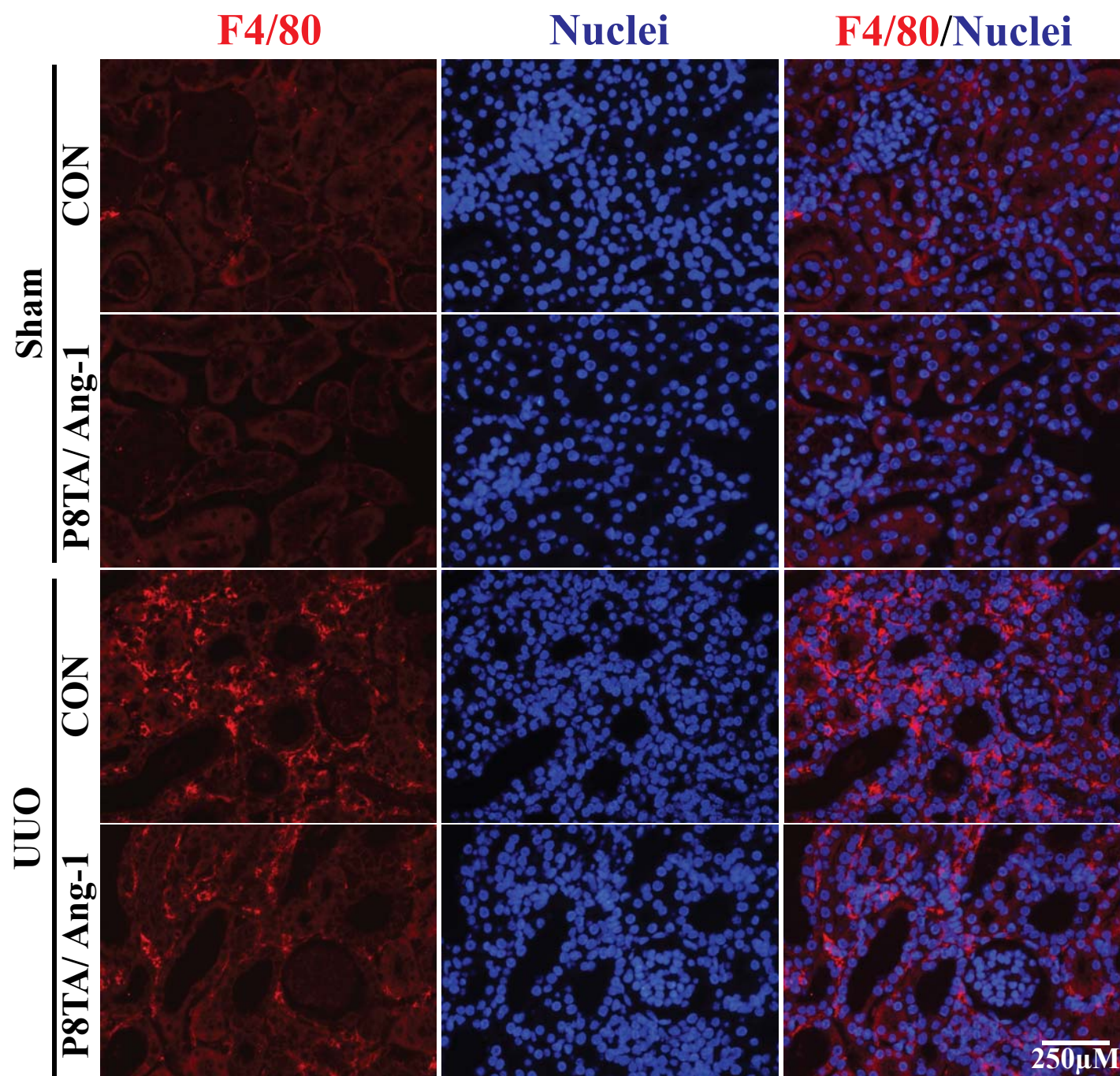

S2 Fig

Supplement: S2 Fig — Nuclei were counterstained with Hoechst 33342 (blue). Individual and merged images were shown as indicated. Scale bar, 250 μm. CON, control. (PDF) [file pone.0158908.s002.pdf]

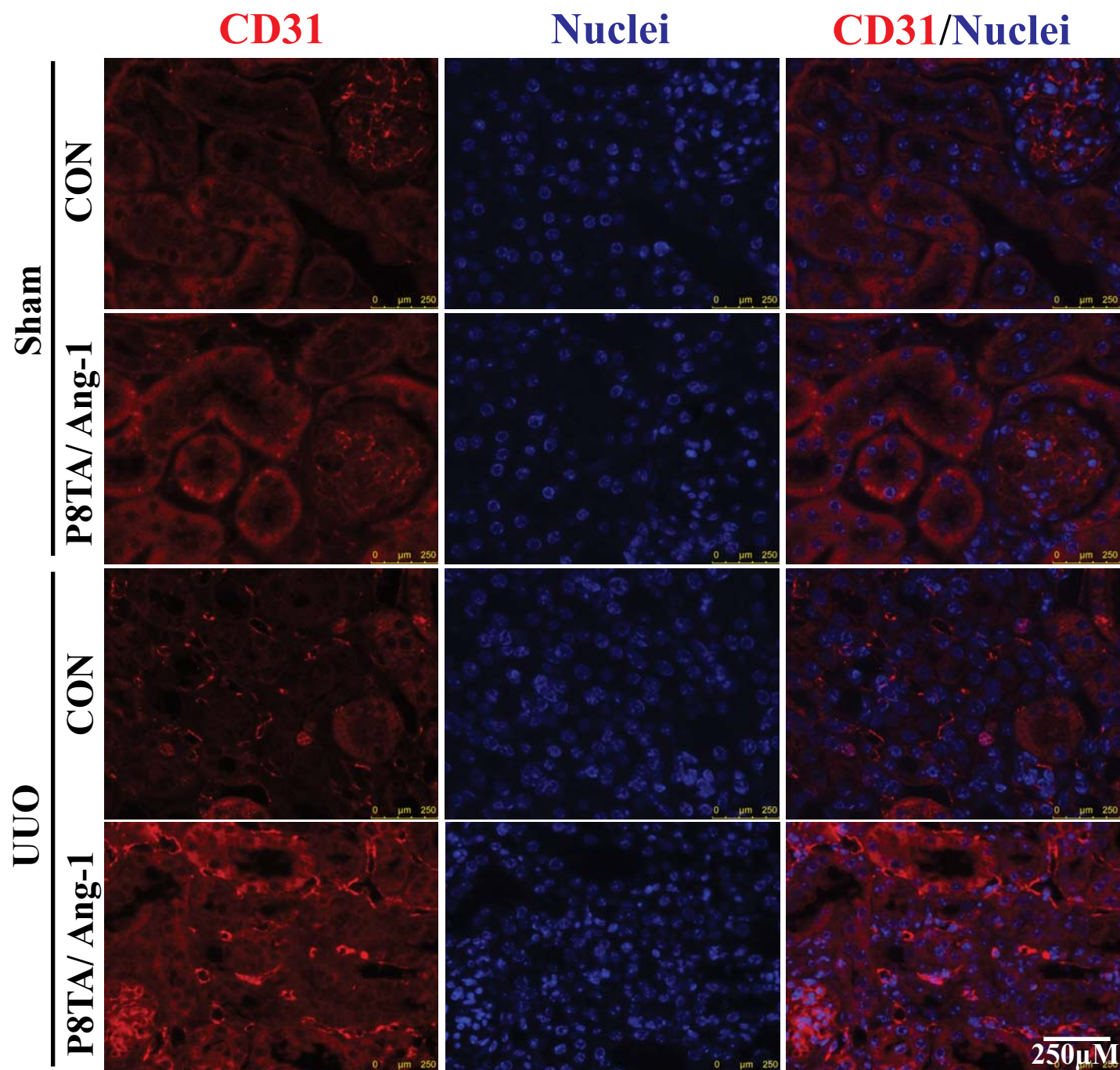

S3 Fig

Supplement: S3 Fig — Nuclei were counterstained with Hoechst 33342 (blue). Individual and merged images were shown as indicated. Scale bar, 250 μm. CON, control. (PDF) [file pone.0158908.s003.pdf]
